# Utilization of day surgery services at Upper hill Medical Centre and the Karen hospital in Nairobi: the influence of medical providers, cost and patient awareness

**DOI:** 10.11604/pamj.2015.22.28.4913

**Published:** 2015-09-11

**Authors:** Mildred Adhiambo Odhiambo, Susan Njuguna, Rachel Waireri-Onyango, Josephat Mulimba, Peter Mungai Ngugi

**Affiliations:** 1 Department of Health Systems Management and Medical Education, Kenya Methodist University, Nairobi, Kenya; 2 Upper Hill Medical Centre, Nairobi, Kenya

**Keywords:** Day surgery, health system, medical providers, medical insurance providers, in patient, patient awareness, cost and utilization

## Abstract

**Introduction:**

Health systems face challenges of improving access to health services due to rising health care costs. Innovative services such as day surgery would improve service delivery. Day surgery is a concept where patients are admitted for surgical procedures and discharged the same day. Though used widely in developed countries due to its advantages, utilization in developing countries has been low. This study sought to establish how utilization of day surgery services was influenced by medical providers, patient awareness and cost among other factors.

**Methods:**

The study design was cross sectional with self administered questionnaires used to collect data. Data analysis was done by using statistical package for social science (SPSS) and presented as frequencies, percentages and Spearman's correlation to establish relationship among variables.

**Results:**

Medical providers included doctors, their employees and medical insurance providers. Most doctors were aware of day surgery services but their frequency of utilization was low. Furthermore, medical insurance providers approved only half of the requests for day surgery. Doctors’ employees were aware of the services and most of them would recommend it to patients. Although, most patients were not aware of day surgery services those who were aware would prefer day surgery to in patient. Moreover, doctors and medical insurance providers considered day surgery to be cheaper than in patient.

**Conclusion:**

The study showed that medical providers and patient awareness had influence over day surgery utilization, though, cost alone did not influence day surgery utilization but as a combination with other factors.

## Introduction

The health system environment is continuously evolving to meet new health challenges. Escalating health care costs and increase in demand for health services are some of the issues hampering access to health services worldwide. The concept of Day surgery has been implemented in developed countries to reduce the cost of surgery and improve access to health services [1]. Day surgery, also referred to as ambulatory surgery is a concept where a patient undergoes surgery and is discharged the same day. First introduced by Dr James Nicoll who performed paediatric surgeries in a free standing Day Surgery Unit (DSU), the concept took several years to be accepted [2]. Day Surgery Units are classified as hospital integrated hospital autonomous, hospital satellite and stand alone or free standing units. A hospital integrated DSU shares the same roof as the rest of the hospital while hospital autonomous is housed within the hospital compound but not under one roof. Conversely, hospital satellite is owned and managed by the hospital but is found in a different location or town while stand alone or free standing DSUs are not affiliated to any hospital [3]. The first DSU established in 1962 in USA was hospital integrated followed by a free standing DSU in 1969. Dramatic increase in DSUs was seen in USA, Canada, UK and Australia between 1970s and 1980s. Increasing numbers of DSUs prompted formation of associations to promote quality standards, expansion, education and research [2]. In Africa slow adoption has been attributed to; poor compliance to bookings, cancellations caused by lack of resources in public institutions, complicated procedures as patients seek medical care late, poor communication and infrastructure that may delay access to emergency care. Moreover, stand-alone DSUs are rare in Africa and more so in rural areas due to fear of not being able to handle emergencies [4, 5].

In Kenya, most private hospitals perform day surgery procedures using the same theatres and recovery wards as inpatients instead of designated Day Surgery Units. The first Integrated DSU was set up at the Nairobi hospital in 1998 but has since changed into a short stay facility that accommodates patients for forty eight hours. Upper Hill Medical Centre Day Surgery Unit was the first stand-alone DSU set up in October 2002 and the trend is being emulated. In spite of the minimal literature on existence of DSU's, they are known to provide support to the Kenyan health system [6]. Day surgery utilization determines revenue collection and viability of the unit. Due to the high costs of managing hospital theatres and DSU, optimum utilization of the operating rooms is a management concern [7]. However, various internal and external factors influence utilization of day surgery services globally. Internal factors focus on management aspects such as scheduling and turn-around time, ensuring optimum utilization of operating rooms. While, external factors include: patient awareness, attitude of doctors and medical insurances towards day surgery and cost of procedures [7]. This study focused on how utilization is influenced by medical providers, cost and patient awareness which are external factors. Day surgery utilization is a health system concern as it directly affects various health system building blocks (pillars). A review of external factors in relation to the pillars revealed that patient awareness determined access as knowledgeable patients would make informed decisions while seeking healthcare services. The attitude of doctors determined how responsive and reactive they were as required by the human resource building block. While, the insurance providers have a responsibility of motivating doctors to improve their services or provide appropriate services by ensuring health services are affordable and accessible. This study was focusing on the service delivery pillar of the health system. Finance and health workforce complement activities to improve service delivery.

## Methods

The study was done at Upper Hill Medical Centre doctor's offices, the Karen Hospital surgical clinics, offices of medical insurance providers and doctors’ clinics in Nairobi, Kenya. The study design was cross-sectional with data being collected at a point in time. The target population included 44 case managers from medical insurances and representatives in companies offering outpatient medical insurance to patients at UHMC and Karen Hospitals; all doctors in Nairobi (2090) from various specialties were targeted to reduce bias; (43) doctors’ employees and (6640) patients from outpatient surgical clinics at Karen Hospital and doctors’ clinics at UpperHill Medical Centre. Cluster sampling was used to determine doctors’ population, stratified sampling to group doctors into various specialties and convenience sampling to identify respondents from the list. Random sampling was used to select patients and doctors’ employees, while, stratified sampling was used for MIPs. All the total of 753 out of 803 sampled responded. Self-administered questionnaires were used to collect the data which took three months. The questionnaires were written in simple language and had standardized questions developed in relation to the objectives. Ethical approval was obtained from Kenya Methodist University's Institutional Research and Ethics Committee (IREC); Ethical committees of Upper Hill Medical Centre and Karen Hospital. Data was analysed as frequencies and percentages. Spearman's correlation was applied to establish relationships among variables and cross tabulation of descriptive data was also done using statistical package for social science (SPSS).

## Results

### Influence by Medical Providers

Medical providers included doctors, medical insurance providers and doctors’ employees.

### Doctors

Doctors were divided into General practitioners (GPs), surgeons and anaesthetists. The study revealed that 195(98.5%) GPs were aware of the services while, 89(92.7%) surgeons and 10(100%) anaesthetists had used the services. Furthermore, 178 (89.9%) GPs were seen to refer patients while only 20 (10.1%) did not. However, most surgeons 51(61.5%) and anaesthetists 5(50%) used day surgery only once a week; 31(32.3%) surgeons and 2(20%) anaesthetist 2-3 times a week; 5(5.2%) surgeons and 2(20%) anaesthetists 4-10 times a week and only 1(1%) surgeon and 1(10%) anaesthetist used the services more than 10 times a week. Patient safety at home, pain control and risk of complications were some of the disadvantages that would discourage utilization; all the doctors were not deterred from using day surgery because of the specific disadvantages. Neither the anaesthetists nor GPs would refuse to use day surgery because of pain though, 3(1.5%) of GPs would be reluctant due to risk of complications. However, when the factors were combined 8(80%) anaesthetists, 93(96.9%) surgeons and 191(96.5%) GPs would be reluctant to use day surgery (Table 1). Factors that encouraged use of day surgery such as reduced cost would influence only 14(7.1%) GPs, 17(17.7%) surgeons and none of the anaesthetists; early ambulation would influence 5(2.5%) GPs while, patient's choice would influence 11(11.5%) surgeons. Very few surgeons 20(20.8%) would use day surgery because it was provided by the hospital while only 1(1%) surgeon would use a particular type of day surgery because it had access to hospital services in case of an emergency after discharge and 6(3%) GPs would prefer day surgery because of comfort of patient recovering with family members at home. However, a combination of the factors would influence 173(87.4%) GPs, 46(47.9%) Surgeons and 10(100%) anaesthetists to use day surgery.

**Table 1:** Factors discouraging doctors from using day surgery services

	Surgeons	Anesthetists	GPs
Patient safety at home	1%	20%	2.0%
Pain control	4.2%	0	0
Risk of complications	2.1%	0	1.5%
Combined factors	92.7%	80%	96.5%

### Medical Insurance providers (MIPs)

Most providers had existed for more than 10years 25(80.6%) compared to 3(9.7%) who had been there between 3-5years and 1(3.2%) for less than 2years. In spite of the long existence for majority of providers only 15(48.4%) of the requested day surgery procedures were approved mainly due to a combination of factors. Individual factors had minimal influence as type of procedure determined only 1(3.2%) approval and lower costs 2(6.5%), though, company policy influenced the decision to approve most 13(41.9%) requests. However, approvals are also influenced by insurance providers 1(3.2%), patients 2(6.5%) and doctors 16(51.6%), while a combination of insurance providers, patients and doctors would influence 12(38.7%) of MIPs to give approval. Amount of money approved for procedures is mainly determined by complexity of procedure 23 (74.2%) but not by doctors fees 3(9.7%) or combined factors 5(16.1%). MIPs would not be discouraged from approving day surgery because of disadvantages such as pain control 1(3.2%); risk of complications 15(16.1%); patient's general condition 1(3.2%) and capacity of medical centre 1(3.2%) but when the factors are combined it would discourage 23(74.2%) of providers. Moreover, factors discouraging utilization would influence approval of day surgery service with p value of 0.426. Medical insurance providers would rather educate surgeons 13(41.9%), than give incentives (19.4%), dictate terms 2(6.5%) or a combination of the factors 10(32.3%) in order to encourage utilization of day surgery services. Spearman's correlation results reveal that determinants are influenced by motivation factors with a p value of 0.396 and approval of day surgery with a p of 0.359. Consequently, utilization of day surgery services may add value to patients and MIPs. According to 1(3.2%) of respondents it would reduce premiums for patients; improve access of healthcare services 17(54.8%); increase number of insured patients 1(3.2%); increase profits for companies 3(9.7%) while, a combination of the factors would influence 9(29%) of respondents. Opinion of MIPs on day surgery would influence approval significantly with a p- 0.360; while individuals influencing day surgery may be influenced by motivation factors p-0.481 (Table 2).

**Table 2:** Correlation results for medical insurance providers

	Motivation factors ( p value)	Approval of day surgery (p value)	Influence on health insurance (p value)
Approval determinants	0.396^+^	0.359^+^	
Factors discouraging utilization		0.416^+^	
Opinion of day surgery		0.360^+^	
Who influences day surgery	0.481^++^		0.399^+^

### Doctors’ Employees

These were nurses 18(39.1%), Secretaries 19 (41.3%); other support staff 9(19.6%). Most of them 45(97.8%) have heard of day surgery services, while only 1(2.2%) has not heard and their main source of information are the doctors 38(82.6%) while others are friends 5(10.9%), newsletter 3(6.5%). Due to the awareness of day surgery services 43(93.5%) of doctors’ employees would recommend the services to patients, however, some of them would be discouraged to use day surgery due to challenges of taking care of operated site at home 2(4.3%); pain at home 3(6.5%); fear of developing complications 33(71.7%), while the three factors combined would only discourage 8(17.4%) of the doctors’ employees. Most doctors’ employees 33(71.7%) are motivated to use day surgery due to reduced cost, recovery at home and early return to work as a combination of factors. While, spearman's correlation results reveal that duration of service of doctors’ employees would influence their referral habits with a high significance of 99.68%; as well as factors that encourage utilization which is significant with a p- value of 0.350.

### Patient Awareness

According to the results, 265(69%) of patients have heard of day surgery and 119(31%) have not, while, 270(70.3%) would opt to use day surgery and 114(29.7%) would opt for inpatient services. Though, 346(90.1%) of patients would recommend day surgery services to others. Most patients 175 (45.6%) would suggest to their doctor about day surgery; while, 6 (1.6%) will suggest to the insurance; 146 (38%) to both the doctor and insurance and only 57 (14.8%) would keep quiet. Although, results from MIPs reveal that only 2(6.5%) of patients would influence MIPs to use day surgery while 16 (51.6%) of doctors would influence the MIPs (Table 3).

**Table 3:** Factors discouraging day surgery utilization among patients

	Percentage%
Taking care of operated site at home	4.3%
Having pain at home	6.5%
Fear of developing complications	71.7%
Combined factors	17.4%

### Cost factors influencing utilization

According to 88(91.7%) of doctors and 28(90.3%) of MIPs day surgery services are cheaper than in patient while, 2(6.5%) of MIPs and 6(6.3%) of doctors say the costs are similar to inpatient and 1(3.2%) of MIP and 2(2.15) of doctors consider day surgery more expensive than in patient. Medical insurance providers had the authority to decide whether to allocate day surgery services under patients’ inpatient or outpatient cover. This study revealed that most approvals 17(54.8%) were done under outpatient cover rather than inpatient 14(45.2%). Among doctors 79 (82.3%) preferred fee for service against 2(6.5%) MIPs while 28(90.3%) MIPs preferred discounted fee for service which was only supported by 16(16.7%) doctors (Table 4). Patients used different ways of paying bills, in this study 183(47.7%) patients paid cash, 51(13.3%) were paid for by the employer and 150 (39.1%) paid through insurance. Patient's level of education had an influence on the source of income, most college 62(16.14%) and university educated patients 74 (19.27%) had their hospital bills paid by the insurance or employers. While, ages between 26-35yrs had the majority employed or running a business; the highest numbers of unemployed were between 18-25 years as they were likely to be going through college.

**Table 4:** Opinion of doctors and MIPs on doctors’ remuneration

	Doctors’ Preference	Preference of MIPs
Fee for service	82.3%	6.5%
Discounted/agreed fee for service	16.7%	90.3%
Capitation	0	3.2%
Salary	1%	0

## Discussion

The The role of medical providers from the study indicated that the doctors were aware of day surgery services, which is contrary to previous studies showing that most countries especially in Africa are still struggling with creating awareness among doctors [3]. Popularity of day surgery has been attributed to its lower costs [1–3] contrary to the results of this study where cost on its own would not influence doctors and medical insurance providers to use day surgery. Medical insurance providers in this study approved only 48.4% of requests unlike providers in USA who give 100% approval to encourage day surgery. In this study lower cost on its own had minimal influence on the doctors [8]. Due to utilization challenges in USA, a decision was made to give incentives to doctors [9]. While, this study found that MIPs in Kenya prefer to educate surgeons rather than give incentives or dictate terms. Study on remuneration revealed that neither doctors nor MIPs would consider capitation or salary as a way of remuneration. Though, insurance providers in other studies preferred to use capitation [10]. Doctors preferred fee for service while MIPs preferred discounted fee for service. This disparity would lead to challenges with approval and day surgery utilization. Despite awareness and willingness to refer patients for day surgery procedures, some of the employees are discouraged by fear of complications. Though, these can be prevented through proper patient selection and having skilled surgeons [1, 11]. Patient awareness was 69% while, 70.3% preferred day surgery which was low compared to a UK study that indicated 80% of patients preferred day surgery [11]. Patients of various age groups and college educated patients paying cash were the highest in number while those paid for by the medical insurance providers were few. This was contrary to proposals by World Health Organization that ways to reduce out of pocket expenditures be sought [12].

## Conclusion

Doctors were aware of day surgery services though they rarely used the services, while medical insurance providers were not approving most requests for day surgery procedures. Most patients were not aware of day surgery services but those who were aware would consider using day surgery rather than in patient services while, lower cost of day surgery services on its own did not influence doctors and medical insurance providers to use day surgery. Medical providers and patient awareness had an influence on utilization of day surgery services. Cost on its own did not influence utilization of day surgery services but in combination with other factors. The study recommends education to all medical providers on day surgery and need for a policy to guide medical insurance provider on utilization of day surgery services. Educating medical insurance providers on the cost saving aspect of day surgery as opposed to inpatient and improving patient awareness on benefits of day surgery to increase utilization of the services.
